# The New Tumour Biomarker *miRNA‐371‐3p* Influences Cisplatin Sensitivity of Testicular Germ Cell Tumour Cell Lines

**DOI:** 10.1111/jcmm.70314

**Published:** 2024-12-20

**Authors:** Richard Weiten, Theadora Engler, Hubert Schorle, Jörg Ellinger, Miriam Saponaro, Abdullah Alajati, Daniel Nettersheim, Isabella Syring‐Schmandke

**Affiliations:** ^1^ Department of Urology and Paediatric Urology University Hospital Bonn Bonn Germany; ^2^ Department of Urology Uro‐Oncology, Robot‐Assisted and Specialized Urologic Surgery University Hospital Cologne Köln Germany; ^3^ Department of Developmental Pathology, Institute of Pathology University Hospital Bonn Bonn Germany; ^4^ Urological Research Laboratory, Translational UroOncology, Department of Urology, Medical Faculty and University Hospital Düsseldorf Heinrich Heine University Düsseldorf Düsseldorf Germany

**Keywords:** cisplatin, *miR‐371*, resistance mechanisms, testicular germ cell tumour

## Abstract

Cisplatin is used to treat a variety of malignancies, including testicular germ cell tumours (TGCTs). Although cisplatin‐based chemotherapy yields high response rates, a subset of patients develop cisplatin resistance, limiting treatment options and worsening prognosis. Therefore, there is a high clinical need for new therapeutic strategies targeting cisplatin‐resistant TGCTs. *MicroRNA‐371a‐3p* (*miR‐371*), the new serum biomarker for TGCTs, shows significantly increased expression in cisplatin‐resistant TGCT cell lines compared to sensitive parental cell lines. However, the functional impact of *miR‐371* on cisplatin sensitivity has not been investigated yet. To evaluate the impact of *miR‐37*1 on cisplatin sensitivity, antagomirs were used to inhibit *miR‐371* expression, resulting in a > 98% decrease in *miR‐371* expression. Cisplatin sensitivity was significantly increased after *miR‐371* inhibition in cisplatin‐resistant and corresponding parental TGCT cell lines, indicating a strongly reduced viability and increased apoptosis after cisplatin treatment in *miR‐371*‐inhibited cells. Our results suggest that *miR‐371* may contribute to the development of cisplatin resistance in TGCTs. Interfering with *miR‐371* expression can increase the cisplatin sensitivity of tumour cells, which may represent a promising approach to improve future therapeutic outcomes in patients with TGCTs, especially those with cisplatin‐resistant disease.

## 1 Introduction

Testicular germ cell tumours (TGCTs) represent the most prevalent cancer among men aged 15–40 years, with an increasing incidence over the past four decades [1]. Cisplatin‐based chemotherapy is frequently used in treating various malignancies, including head and neck, lung, gastrointestinal tract and genitourinary cancers, for example, urothelial, vulvar, penile squamous cell carcinoma, as well as ovarian and testicular germ cell tumours [2]. Despite the high efficacy of cisplatin‐based regimens in TGCTs, a subset of patients (~10%) develops cisplatin resistance [1, 3], leading to disease recurrence and poorer prognosis [1]. Moreover, even patients who achieve successful outcomes with cisplatin therapy may face acute and lifelong toxicities [1, 4]. Therefore, there is an urgent clinical need to develop new treatment approaches that address cisplatin refractory TGCTs while minimising the cytotoxic impact of cisplatin.

The molecular mechanisms leading to cisplatin resistance in TGCTs are likely multifactorial and can be categorised into four primary mechanisms [5, 6]: (i) decreased cellular import and increased export of cisplatin (pretarget); (ii) decreased accumulation of cisplatin in the cell (on‐target); (iii) reduced induction of apoptosis (posttarget) and (iv) activated compensatory signalling pathways that antagonise the cisplatin resistance mechanism without being a direct target of it (off‐target) [5, 6].

Previous research has highlighted the upregulation of microRNAs (miRs) from the *miR‐371‐3* and miR‐302/367 cluster across various cancers, particularly in TGCTs, where they were first proposed as new biomarkers in 2011 [7]. These miRs exhibit superior sensitivity and specificity compared to the established serum tumour markers, including beta‐human chorionic gonadotropin, alpha‐fetoprotein and lactate dehydrogenase [8, 9, 10, 11]. In general, miRs are small noncoding RNAs involved in the epigenetic regulation of gene expression and various processes of tumour progression, including proliferation, angiogenesis, epithelial‐to‐mesenchymal transition, metastasis and DNA repair [12, 13]. Among them, *miR‐371‐3p* (*miR‐371*) has emerged as the most sensitive (90.1%) and specific (94.0%) biomarker for TGCT diagnosis, treatment monitoring and detection of residual or recurrent disease [8, 9, 10, 11]. Importantly, *miR‐371* is expressed in both seminomas and nonseminomas, except teratomas [8, 9, 10, 11].

This study aimed to investigate the influence of *miR‐371* on cisplatin sensitivity in parental TGCT cell lines and their matched cisplatin‐resistant subclones, thereby enhancing our understanding of the molecular basis of cisplatin response and resistance mechanisms.

## 2 Materials and Methods

### 2.1 Cell Lines and Culture Conditions

Human parental TGCT cell lines, NCCIT, 2102EP and NT2/D1 (arises from an embryonic carcinoma) and their respective cisplatin‐resistant subclones, NCCIT‐R, 2102EP‐R and NT2/D1‐R (provided with the generous permission of Oing C. et Honecker F. [14, 15]) were grown in a 5% CO_2_ incubator at 37°C. Cisplatin‐resistant subclones were generated by cultivating the parental cell lines in increasing concentration of cisplatin as published [14, 15, 16]. Monolayer cultures were maintained in RPMI 1640 medium (#31870‐025; Thermo Fisher Scientific, Darmstadt, Germany) supplemented with 10% heat‐inactivated foetal calf serum (#26140‐079; Thermo Fisher Scientific), 0.8% streptomycin–penicillin antibiotics (10,000 units/mL penicillin and 10,000 μg/mL streptomycin) (#15140‐122; Thermo Fisher Scientific) and 1% l‐glutamine (200 mM; #25030‐024; Thermo Fisher Scientific).

### 2.2 RNA Transfection and RNA Isolation

By using two different antagomiRs, miRCURY LNA miRNA inhibitor HSA‐MIR‐371A‐3P (#267258255; Qiagen, Hilden, Germany, inhibitor 1) and has_miRNA inhibitor (#76209062; IDT, Iowa, USA, inhibitor 2), we established a *miR‐371*‐inhibition. The miRCURY LNA miRNA inhibitor control (Negative control A [#201802070018‐2; Qiagen]) was used as miR‐inhibitor negative control (CTRL). Tumour cells were transfected with 100 μL transfection mix (10% inhibitor/negative control 10 μM solution, 10% HD‐transfection reagent FuGENE) (#E2311; Promega Corporation, Madison, USA, 90% RPMI) in 1.9 mL culture medium. After incubating the cells for 48 and 72 h, RNA was isolated from cell line pellets using the Total RNA Purification Mini Spin Column Kit (Genaxxon Bioscience GmbH, Ulm, Germany), and RNA quantity and quality were measured using the NanoDrop 2000 spectrophotometer (Thermo Fisher Scientific).

### 2.3 Quantitative Reverse Transcriptase‐Polymerase Chain Reaction

Complementary DNA (cDNA) was synthesised using the miScript II RT Kit (#218161; Qiagen). Quantitative reverse transcriptase‐polymerase chain reaction (qRT‐PCR) was performed using 5 ng/mL cDNA and miScript SYBR Green PCR Kit (#218073; Qiagen). The following predesigned Qiagen miScript primer sequences were used: Hs_miR‐371_1 (MS00004060), Hs_miR‐335_1 (MS00003976) and Hs_RNU1A_11 (MS00013986). All samples were run in triplicates, and the relative expression was calculated using the equation RQ = 2^−ΔΔCT^.

### 2.4 Measurement of Cell Viability

To measure the cisplatin sensitivity after miR‐371‐inhibition (performed with inhibitor 1), we used a Crystal violet assay. The assay involves exposing the cells (8 × 10^3^ or 1 × 10^4^ cells/well) to different concentrations of cisplatin (0–12 μM) for 24–72 h. Triplicates were made for all conditions. For staining, cells were fixed with 37% paraformaldehyde for 10 min, washed with distilled water and stained with 0.05% crystal violet for 30 min. Cells were washed with distilled water again and dried in room air. To dissolve the dye, 0.1% acetic acid per well was added. The absorbance of the stained cells was measured using an ultraviolet–visible spectrometer (570 nm, Safire Reader [Tecan]). The viability of the cells was calculated based on the absorbance values.

### 2.5 Flow Cytometry

Flow cytometry was used to analyse the rates of apoptosis (programmed cell death) after miR‐371‐inhibition (performed with inhibitor 1) by using Annexin V/propidium iodide (PI). The staining procedure was carried out according to standard protocols. Early apoptotic cells were defined as Annexin V‐positive/PI‐negative, whereas late apoptotic cells were defined as Annexin V/PI‐positive and necrotic cells were Annexin V‐negative/PI‐positive. Viable cells remained unstained (Annexin V/PI‐negative). A total of 5 × 10^4^ cells was measured for each sample. Single‐cell suspensions of NCCIT and 2102EP and their respective cisplatin‐resistant subclones were stained with 5 μL Annexin V (#640919; BioLegend, San Diego, CA, USA) followed by 20 μg/mL PI (#421301; BioLegend). Data were acquired using a Cytec Aurora flow cytometer (Cytek Biosciences, Fremont, CA, USA) and analysed with the FlowJo software (FlowJo v10.8; BD, https://www.flowjo.com).

### 2.6 Statistical Analysis

Statistical analysis was conducted using GraphPad Prism (Version 9.4.0). The parametric *t*‐test was used to statistically compare two groups, while the parametric one‐way ANOVA was employed to compare multiple groups. All *p‐*values were calculated two‐sided, and *p* < 0.05 was considered statistically significant.

## 3 Results

### 3.1 *miR‐371* Is Overexpressed in TGCT Cisplatin‐Resistant Cell Lines

**FIGURE 1:**
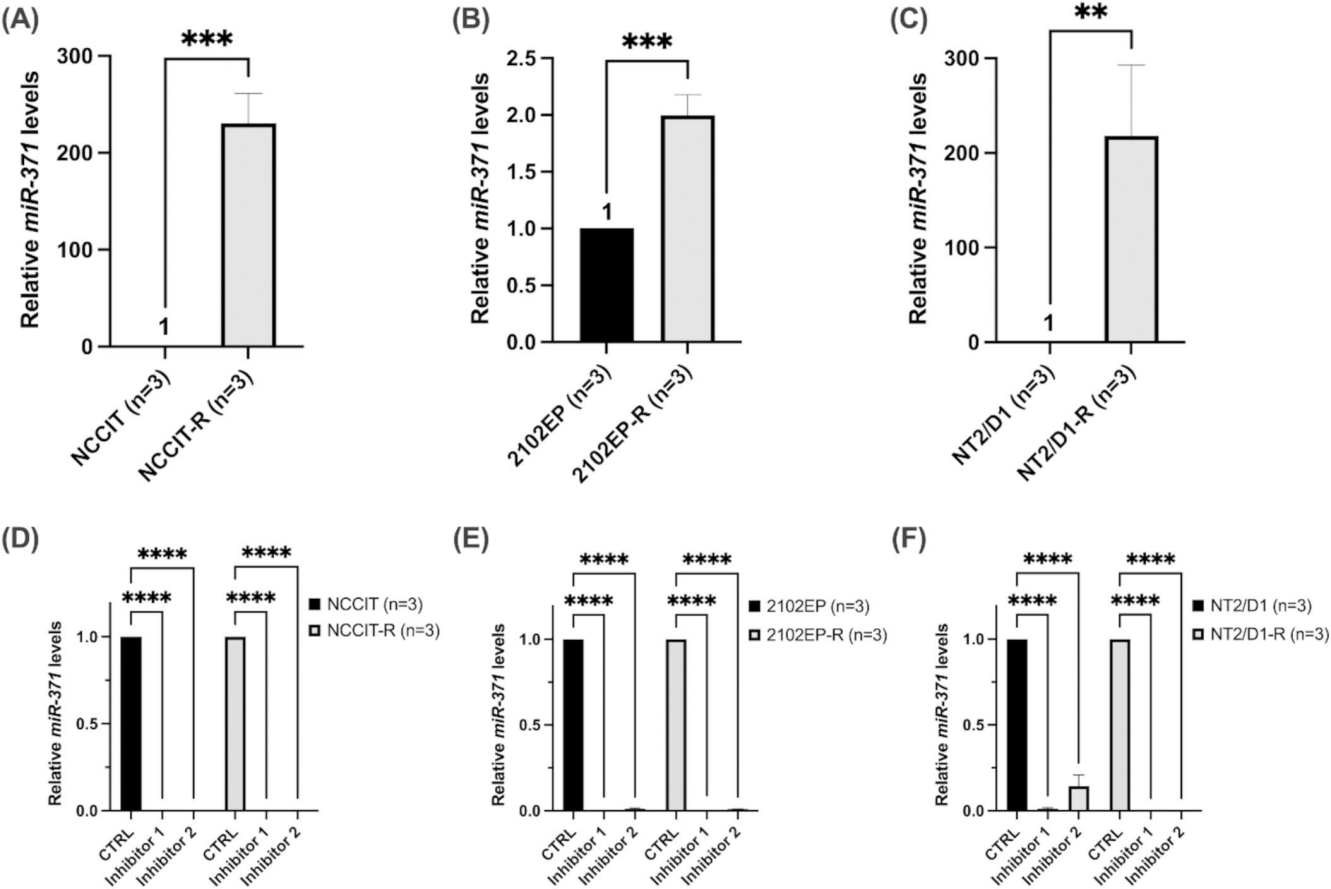
(A–C) Expression analysis of *miR‐371* in TGCT cell lines. *miR‐371* expression was significantly increased in cisplatin‐resistant testicular germ cell tumour (TGCT) cell lines (NCCIT‐R, 2102EP‐R and NT2/D1‐R) compared to sensitive parental cell lines (NCCIT, 2102EP and NT2/D1). (D–F) Downregulation of *miR‐371* levels in TGCT cell lines. By using two different antagomirs (Inhibitors 1 and 2), the inhibition of *miR‐371* led to a reduced *miR‐371* expression (> 98%) in all parental and corresponding cisplatin‐resistant TGCT cell lines. The relative fold change of *miR‐371* expression was determined using the equation RQ = 2^−ΔΔCT^ (unpaired *t*‐test, **p < 0.01, ***p < 0.001, ****p < 0.0001).

In three nonseminomatous TGCT cell lines (NCCIT, 2102EP and NT2/D1) and their matched cisplatin‐resistant subclones (NCCIT‐R, 2102EP‐R and NT2/D1‐R), the expression levels of *miR‐371* were determined by qRT‐PCR. The *miR‐371* expression was significantly increased in the cisplatin‐resistant TGCT cell lines compared to sensitive parental cell lines (230.6 ± 30.79 [NCCIT vs. NCCIT‐R], 1.997 ± 0.178 [2102EP vs. 2102EP‐R], 218.2 ± 74.84 [NT2/D1 vs. NT2/D1‐R], *p* < 0.01), as depicted in Figure 1A–C. These data represent an initial indication of the potential involvement of *miR‐371* in the development of cisplatin resistance in TGCT cell lines.

### 3.2 Enhanced Cisplatin Sensitivity After *miR‐371* Downregulation in TGCT Cell Lines

To assess the functional impact of *miR‐371* on cisplatin sensitivity in TGCT cell lines, we inhibited *miR‐371* by using two different antagomirs (inhibitors 1 and 2), leading to a decreased *miR‐371* expression in the cisplatin‐resistant and corresponding parental TGCT cell lines (NCCIT, 2102EP and NT2/D1). A significant *miR‐371* downregulation of over 98% was observed in all TGCT cell lines (0.0011 ± 0.0004 [NCCIT], 0.0059 ± 0.0069 [2102EP], 0.0835 ± 0.0780 [NT2/D1], 0.0001 ± 0.0002 [NCCIT‐R], 0.0045 ± 0.0044 [2102EP‐R], 0.0006 ± 0.0006 [NT2/D1‐R], *p* < 0.0001) with at least one antagomir, as illustrated in Figure 1D–F.

**FIGURE 2:**
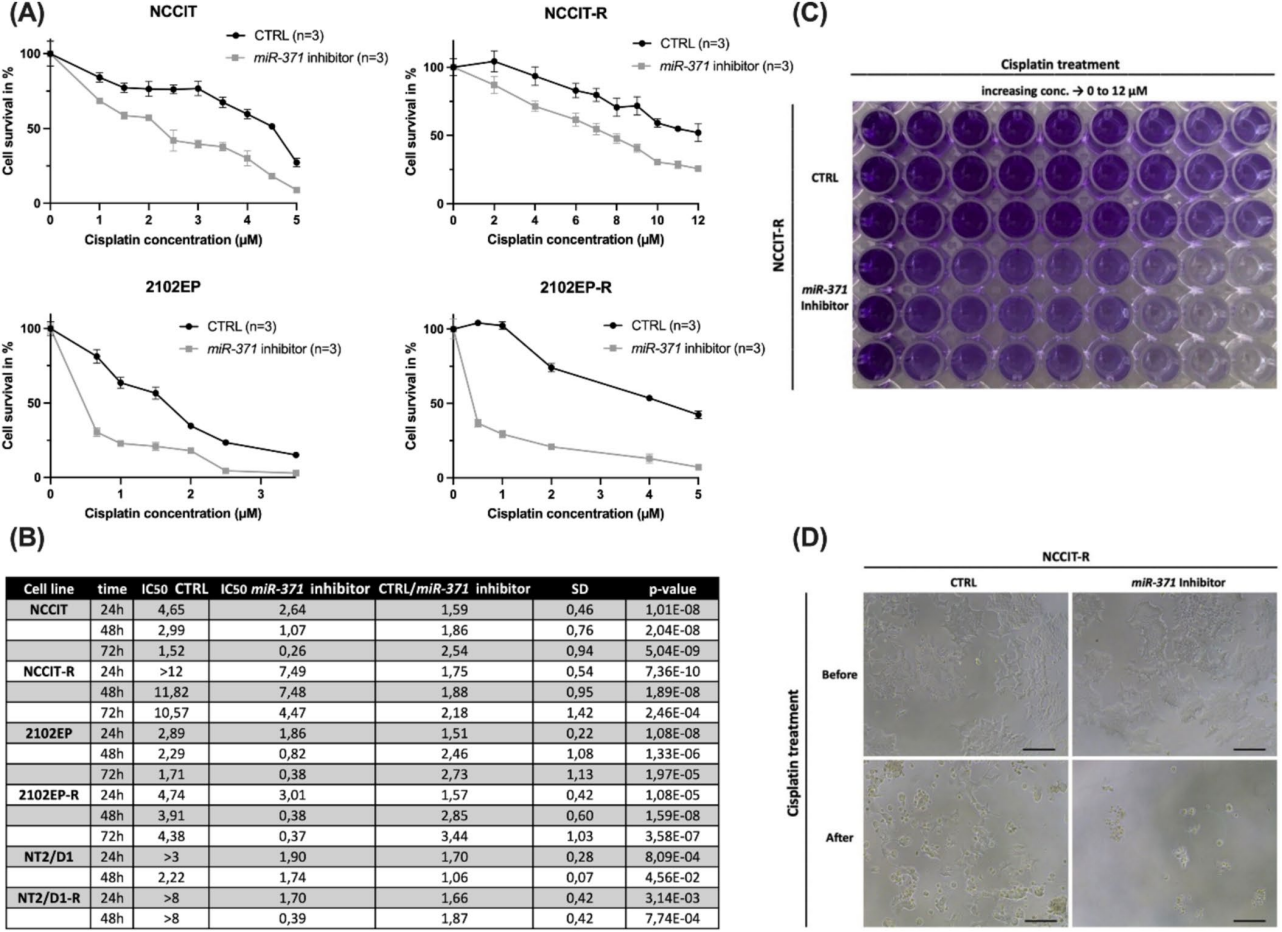
Enhanced cisplatin sensitivity in testicular germ cell tumour (TGCT) cell lines after *miR‐371‐*downregulation. (A, B) The IC_50_ was determined for each TGCT cell line (NCCIT, 2102EP, NCCIT‐R, 2102EP‐R). *miR‐371*‐inhibited and negative control cells were exposed to different concentrations of cisplatin (0–12 μM) for 24–72 h. After *miR‐371* inhibition, the cisplatin sensitivity was significantly increased, indicated by a strongly reduced viability after cisplatin addition in *miR‐371*‐inhibited cells as compared to control cells. (C, D) Representative macroscopic and microscopic images (10× and 40× magnification) of the different appearances of control and *miR‐371* inhibited cells (NCCIT‐R). CTRL, miR‐inhibitor negative control.

For drug sensitivity analysis, *miR‐371*‐inhibited and control cells were exposed to different concentrations of cisplatin (0–12 μM) for 24–72 h. For this purpose, the IC_50_ was first determined for each parental and corresponding cisplatin‐resistant TGCT cell line. After the addition of different cisplatin concentrations in the range of the IC_50_, changes in the viability of the cells were measured by crystal violet assays. Cisplatin sensitivity was significantly increased after the *miR‐371* inhibition, indicated by a strongly reduced viability after cisplatin addition in *miR‐371*‐inhibited cells as compared to control cells (*p* < 0.001) (Figure 2A–D). Representative microscopic images of the different appearances of control and *miR‐371*‐inhibited TGCT cells (NCCIT, NCCIT‐R, 2010EP, 2102EP‐R) are illustrated in Figure S1. The decrease in cell viability correlated with the duration and concentration of cisplatin treatment, indicating that *miR‐371* inhibition may be a promising strategy for enhancing the effectiveness of cisplatin in the treatment of TGCTs.

### 3.3 Enhanced Apoptosis Rate After *miR‐371* Downregulation in TGCT Cell Lines

**FIGURE 3:**
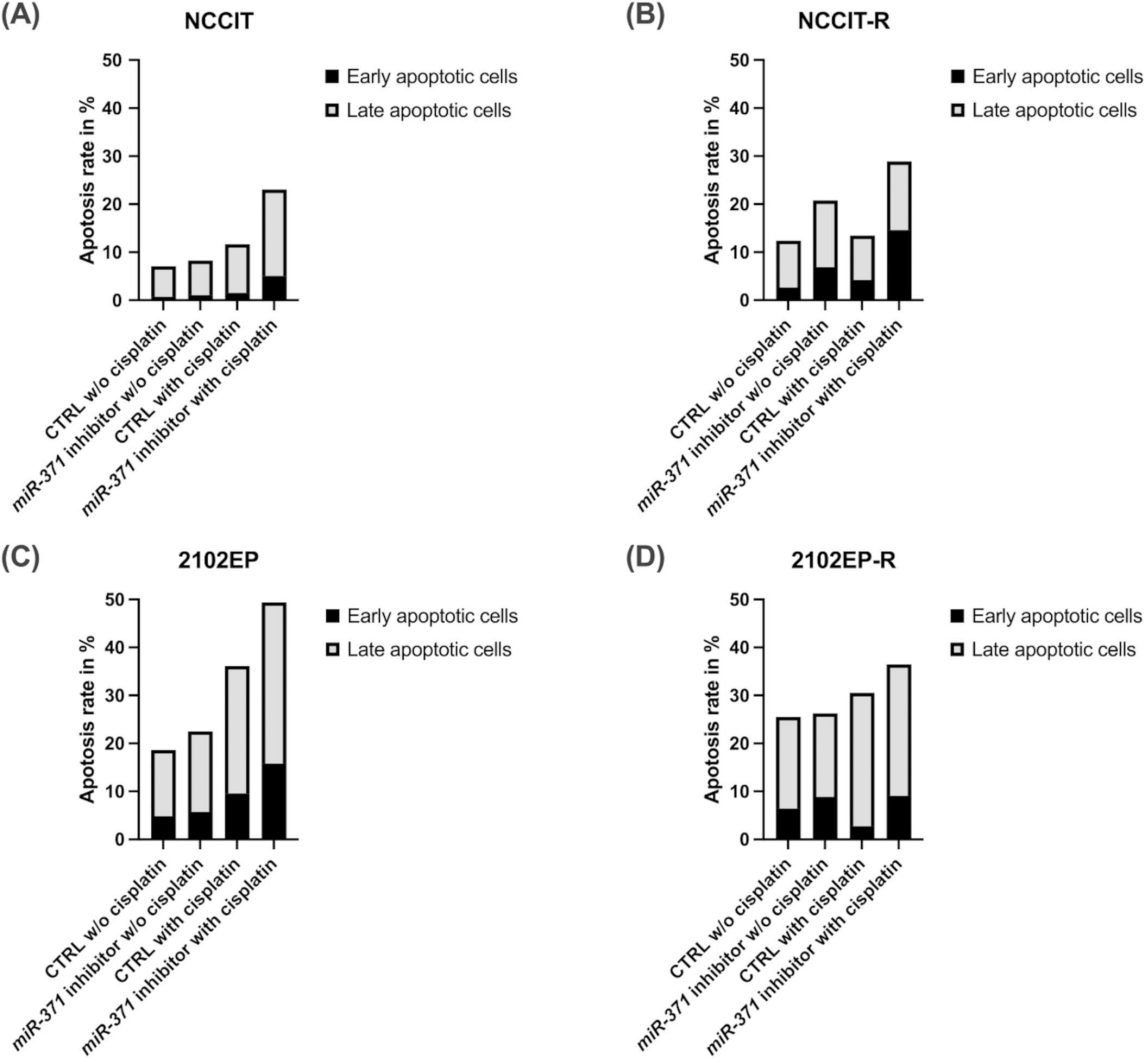
Increased apoptosis rate in testicular germ cell tumour (TGCT) cell lines after *miR‐371‐*downregulation. (A–D) Flow cytometry assay (Annexin V/PI) was used to measure the apoptotic rates induced by the downregulation of *miR‐371*. In cisplatin‐resistant, (NCCIT‐R, 2102EP‐R) and sensitive parental (NCCIT, 2102EP) TGCT cell lines, cisplatin sensitivity was significantly enhanced after the *miR‐371* inhibition, indicated by increased apoptosis after cisplatin application in *miR‐371*‐inhibited cells compared to miR‐negative control cells (1.669 ± 0.460). CTRL, miR‐inhibitor negative control.

To assess the apoptotic rates induced by the downregulation of *miR‐371*, we employed a flow cytometry assay (Annexin V/PI). In the cisplatin‐resistant and corresponding parental TGCT cell lines, cisplatin sensitivity was significantly raised after the *miR‐371* inhibition, as evidenced by increased apoptosis following cisplatin treatment in *miR371*‐inhibited cells compared to control cells (1.669 ± 0.460) (Figure 3A–D; Figure S2). The cisplatin concentration used for each cell line was selected based on their respective IC_50_ values. Specifically, *miR‐371* downregulation led to an increase in the percentage of apoptotic cells in the sample, particularly in the early apoptosis population (1.657 ± 0.631), which was further augmented upon cisplatin addition (2.943 ± 0.863). Our data show that apoptosis is increased after cisplatin treatment in *miR‐371*‐inhibited cells.

Analysis of the cell cycle showed no significant differences between *miR‐371*‐treated and control cells (Figure S3). These findings suggest that *miR‐371* downregulation does not significantly impact the cell cycle, but might influence the development of cisplatin resistance.

## 4 Discussion

Despite advancements in cancer treatments, the mechanisms underlying cisplatin responsiveness and resistance in TGCTs remain poorly understood [4]. Therefore, there is an unmet need for innovative therapeutic approaches to address cisplatin‐resistant TGCTs. Recent studies have reported upregulation of *miR‐371‐3* and *miR‐302/367* clusters in TGCTs [8, 9, 10, 11], with *miR‐371* emerging as the most sensitive and specific biomarker for disease diagnosis, surveillance and recurrence detection [8, 9, 10, 11]. Moreover, increased evidence linked *miR‐371* to the epigenetic regulation of gene expression and cisplatin sensitivity [12].

In this report, we demonstrated for the first time that inhibition of *miR‐371* significantly enhances cisplatin sensitivity in both cisplatin‐resistant and parental TGCT cell lines. Specifically, *miR‐371*‐inhibited cells exhibited decreased viability following cisplatin treatment, while cell viability remained unchanged in the absence of cisplatin, regardless of *miR‐371* status. Additionally, a significantly higher proportion of cells underwent apoptosis upon cisplatin exposure when *miR‐371* was inhibited. These findings suggest that *miR‐371* may contribute to the acquisition of cisplatin resistance and serve as a potential therapeutic target to improve cisplatin efficacy in TGCTs.

Existing literature highlights DNA repair mechanisms and impaired apoptosis as key contributors to cisplatin resistance in TGCT patients [4, 17]. Unlike many other tumours, TGCTs rarely harbour TP53 mutations, though such alterations may occur in mediastinal TGCTs, which may also involve mutations in the RAS pathway [18, 19]. While extragonadal pure seminomas demonstrate similar overall survival rates regardless of primary site, retroperitoneal nonseminomas exhibit higher 5‐year overall survival rates compared to their mediastinal counterparts, emphasising the impact of tumour microenvironment on cisplatin sensitivity [20]. Importantly, TP53 alterations have predominately been observed in cisplatin‐resistant TGCT patients [3]. Increased expression or amplification of MDM2 and MDM4, negative regulators of p53, has been associated with more aggressive and therapy‐resistant TGCT phenotypes [21]. Therefore, treatment with the MDM inhibitor Nutlin‐3 has been shown to significantly elevate TP53 expression, thereby activating p53‐dependent proapoptotic pathways [21]. In contrast, nonfunctional TP53 is present in approximately 95% of ovarian carcinoma cases, contributing substantially to cisplatin resistance due to defective apoptosis induction [22]. In renal cell carcinoma cell lines, CDKN1A knockdown increased p53 protein levels and sensitised cells to cisplatin‐induced apoptosis via p53 [23]. Furthermore, Voorhoeve et al. [24] demonstrated that the *miR‐371‐3* cluster neutralises p53 function in TGCTs by targeting the tumour suppressor LATS2. Thus, *miR‐371* inhibition may enhance cisplatin sensitivity through the restoration of p53‐mediated apoptotic pathways.

In the context of precision medicine, identifying tumour‐specific aberrations that dive into oncogenic growth and resistance through activation of oncogenes or suppression of tumour suppressor genes, including epigenetic modifications, has facilitated the evaluation of targeted therapies. The combination of nonselective chemotherapy with agents targeting resistance‐mediating pathways may improve therapeutic efficacy and reduce adverse effects [25]. Modulating *miR‐371* expression in combination with targeted cancer therapies may offer a promising approach to delaying or overcoming acquired cisplatin resistance. Given the overall high cisplatin sensitivity of TGCTs, integrating platinum‐sensitising agents with platinum‐based regimens may overcome resistance in challenging clinical cases, while potentially reducing chemotherapeutic dosages in responsive patients to minimise acute and long‐term toxicities [25]. Importantly, miR‐371 inhibition not only increases cisplatin sensitivity in resistant tumours but may also enhance sensitivity in cisplatin‐responsive tumours, offering an avenue to reduce chemotherapy doses and associated toxicities [16].

Limitations of this study include the challenge of translating in vitro findings into clinical practice, particularly the exclusive use of embryonal carcinoma cell lines, which limits the generalisation of results across the broader spectrum of TGCTs, including yolk sac tumours and choriocarcinoma. Furthermore, the study focused solely on *miR‐371‐3p*, without evaluating the potential contributions of other miRNAs within the *miR‐371‐3* and *miR‐302/367* clusters. Our methodology emphasised miRNA inhibition but did not include the use of miRNA mimics, which could have provided insights into the opposing effects of *miR‐371* on cisplatin sensitivity. A significant challenge to the clinical application of miRNA inhibitors (‘antimiRs’) remains the risk of severe off‐target toxicities. Consequently, further investigations are required, including in vitro studies utilising a more diverse array of TGCT cell lines, as well as comprehensive in vivo and clinical studies, to thoroughly evaluate the safety and therapeutic potential of miRNA inhibitors.

## 5 Conclusion

In conclusion, our results suggest that *miR‐371* may contribute to the development of cisplatin resistance in TGCTs, and especially downregulation of *miR‐371* expression may increase the cisplatin sensitivity of tumour cells. This may represent a promising approach to improve therapeutic outcomes in patients with TGCTs, especially those with cisplatin‐resistant disease.

## Author Contributions

**Richard Weiten:** conceptualization (equal), data curation (equal), formal analysis (equal), methodology (equal), project administration (equal), software (equal), supervision (equal), visualization (equal), writing – original draft (equal). **Theadora Engler:** data curation (equal), formal analysis (equal), investigation (equal), methodology (equal), writing – original draft (equal). **Hubert Schorle:** resources (equal), writing – review and editing (equal). **Jörg Ellinger:** resources (equal), supervision (equal), writing – review and editing (equal). **Miriam Saponaro:** investigation (supporting), methodology (supporting), writing – review and editing (equal). **Abdullah Alajati:** resources (supporting), writing – review and editing (equal). **Daniel Nettersheim:** conceptualization (equal), methodology (equal), project administration (equal), supervision (equal), writing – review and editing (lead). **Isabella Syring‐Schmandke:** conceptualization (equal), project administration (equal), resources (equal), supervision (equal), writing – review and editing (lead).

## Conflicts of Interest

The authors declare no conflicts of interest.

## Supporting information


Figures S1–S3.

## Data Availability

The data that support the findings of this study are available from the corresponding author upon reasonable request.
