# Supplementary material for: The New Tumour Biomarker miRNA‐371‐3p Influences Cisplatin Sensitivity of Testicular Germ Cell Tumour Cell Lines
Source: J Cell Mol Med. 2024 Dec 20;28(24):e70314. doi: 10.1111/jcmm.70314 (PMC11661915; doi:10.1111/jcmm.70314)
Supplement: Supplementary file 1 — Figures S1–S3. [file JCMM-28-e70314-s001.docx]

**
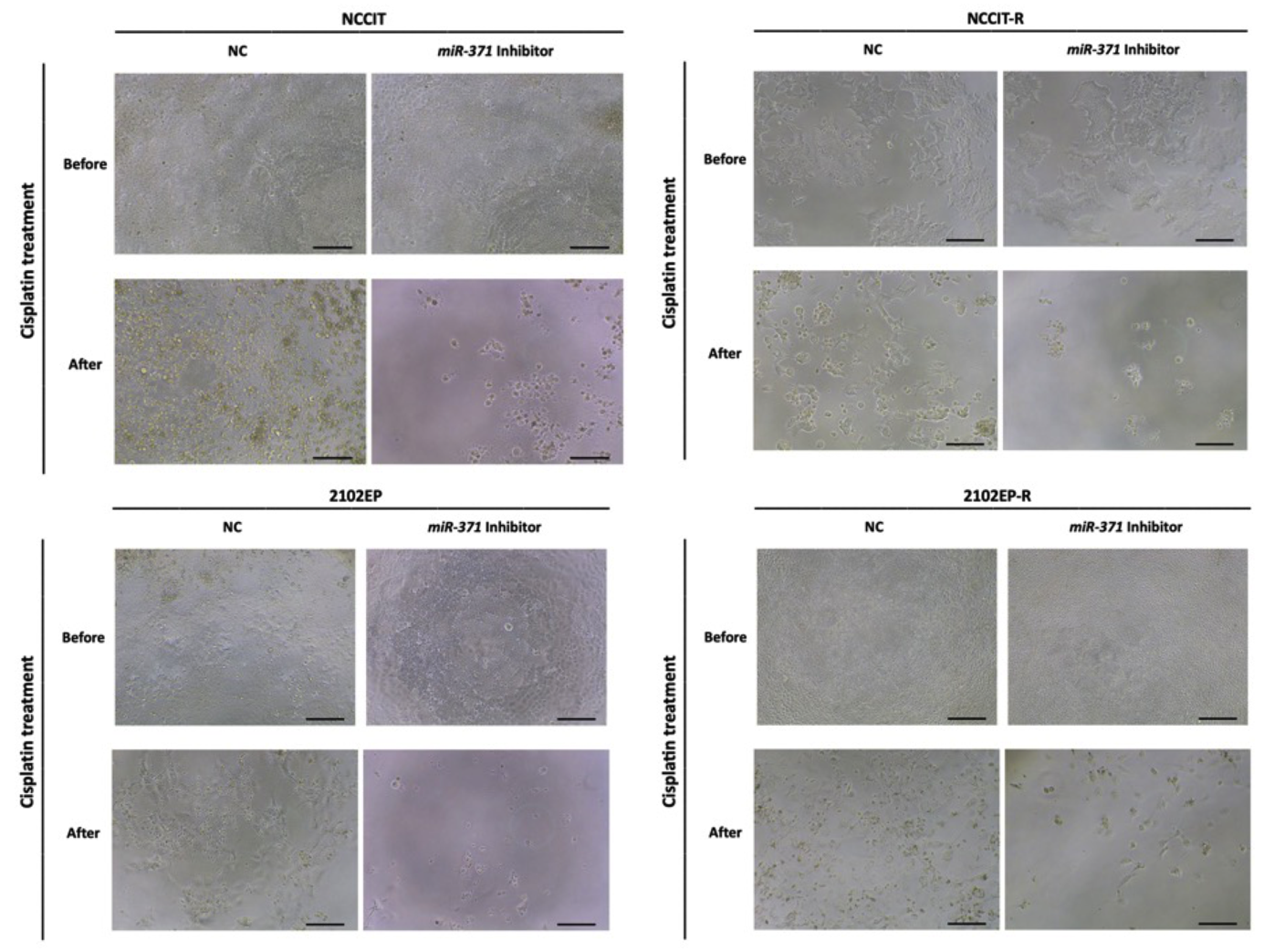
Supporting information**

**Figure S1**. **Enhanced cisplatin sensitivity in testicular germ cell tumor (TGCT) cell lines after *miR-371-*downregulation**. Representative microscopic images (10x and 40x magnification) of the different appearance of control and *miR-371*-inhibited cells in all TGCT cell lines (NCCIT, NCCIT-R, 2010EP, 2102EP-R). CTRL, miR-371 negative control.


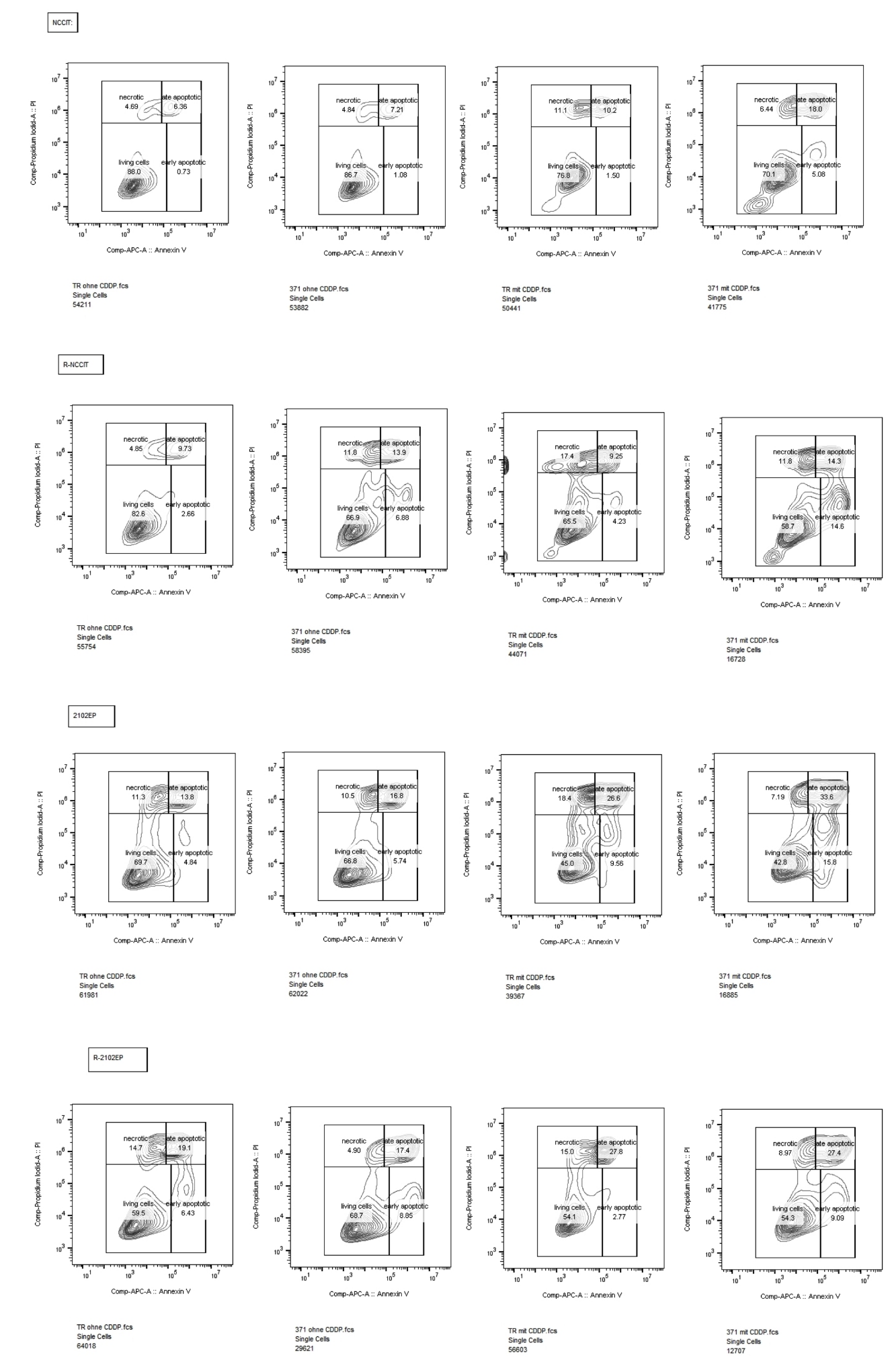
**Figure S2**. Enhanced apoptosis rate in testicular germ cell tumor (TGCT) cell lines after *miR-371-*inhibition in flow cytometry assay.

**
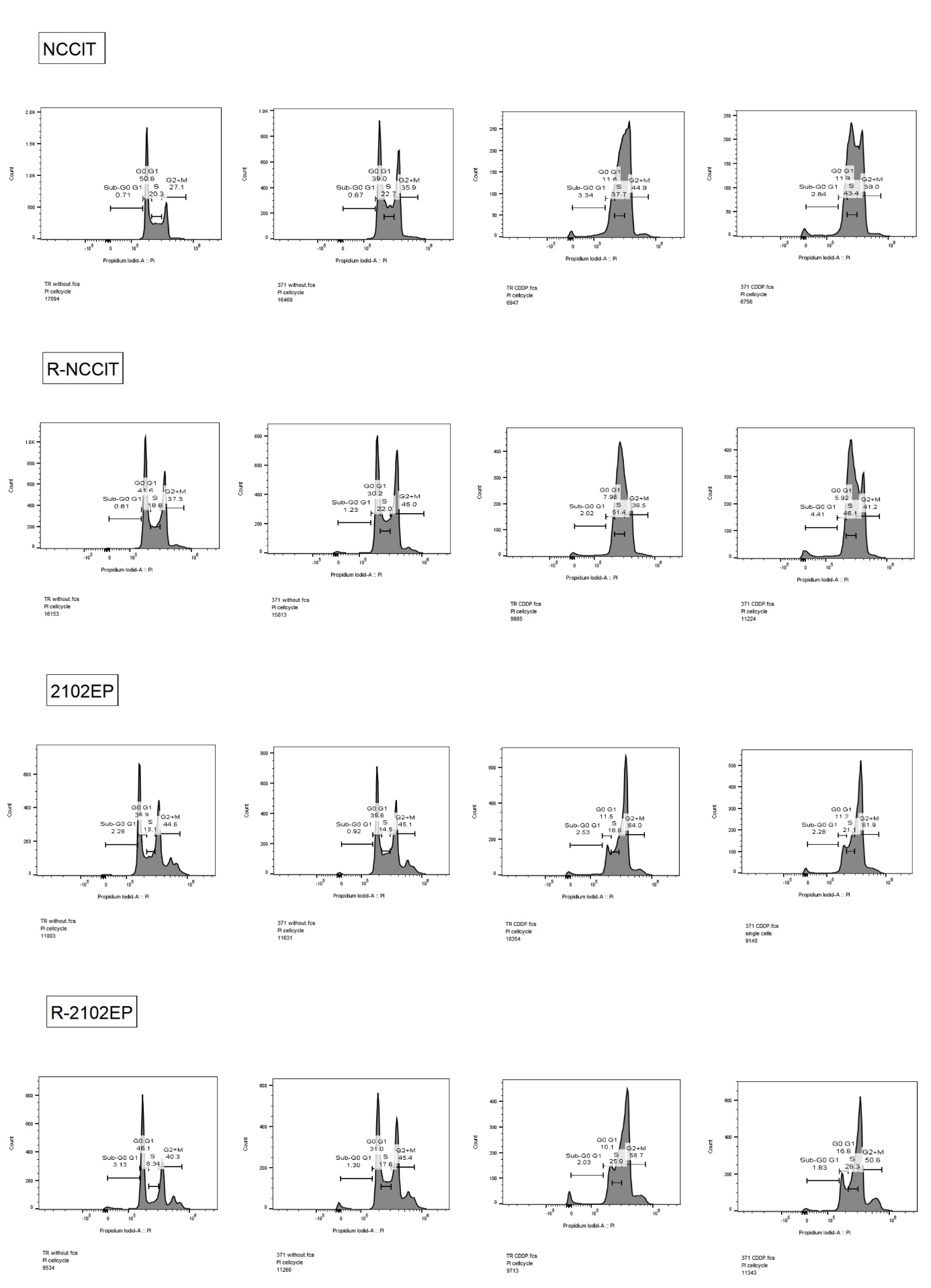
Figure S3**. Cell cycle analysis in testicular germ cell tumor (TGCT) cell lines after *miR-371-*inhibition.
